# Associations between adolescents’ oral health and health literacy, gender and family affluence: perspective of the Health Behaviour in School-aged Children study data from Slovakia and Poland

**DOI:** 10.3389/fpubh.2025.1603356

**Published:** 2025-11-19

**Authors:** Karolina Kamila Glińska, Peter Kizek, Andrea Madarasova Geckova, Zuzana Boberova, Anna Dzielska, Dorota Kleszczewska, Erik Sigmund, Veronika Jurkova, Jaroslava Kopcakova

**Affiliations:** 1 Department of Stomatology and Maxillofacial Surgery, Faculty of Medicine, P. J. Safarik University in Kosice, Kosice, Slovakia; 2 Department of Health Psychology and Research Methodology, Faculty of Medicine, P. J. Safarik University in Kosice, Kosice, Slovakia; 3 Institute of Biology and Ecology, Faculty of Science, P. J. Safarik University in Kosice, Kosice, Slovakia; 4 Department of Child and Adolescent Health, Institute of Mother and Child, Warsaw, Poland; 5 Institute of Mother and Child Foundation, Warsaw, Poland; 6 Department of Health Sociology, Education and Medical Communication, Institute of Mother and Child, Warsaw, Poland; 7 Faculty of Physical Culture, Institute of Active Lifestyle, Palacký University Olomouc, Olomouc, Czechia; 8 Lifelong Learning Centre and Projects Support, P. J. Safarik University in Kosice, Kosice, Slovakia; 9 Medical Education Centre, Faculty of Medicine, P. J. Safarik University in Kosice, Kosice, Slovakia; 10 Olomouc University Social Health Institute, Palacký University Olomouc, Olomouc, Czechia

**Keywords:** oral health, toothbrushing, adolescents, health literacy, family affluence, gender

## Abstract

**Objectives:**

The aim of this study was to examine the association between oral health and health literacy, gender, age, family affluence and country of origin amongst adolescents from Slovakia and Poland, using data from the Health Behaviour in School-aged Children study.

**Methods:**

We analysed data from the cross-sectional Health Behaviour in School-aged Children study conducted in 2022 on a representative sample of 6,289 Slovak and Polish 13- and 15-year old adolescents (mean age 14.48; SD = 1.01; 50.5% boys). Data was collected through self-administered online questionnaires completed by respondents in schools during classes. Binomial logistic regression models were used to assess associations between oral health and health literacy, gender, age, family affluence and country of origin amongst adolescents from Slovakia and Poland.

**Results:**

The results indicate that boys (odds ratio/95% confidence interval OR/95% CI 0.431/0.381–0.489) are substantially less likely to engage in regular toothbrushing compared to girls, highlighting a persistent gender disparity in oral hygiene behaviour. Additionally, lower socioeconomic status, as measured by family affluence, is associated with a decreased likelihood of frequent toothbrushing (OR/95% CI 0.486/0.399–0.592 for low family affluence; OR/95% CI 0.761/0.647–0.895 for medium family affluence). Similarly, health literacy emerges as a key determinant, with adolescents exhibiting lower health literacy levels demonstrating significantly reduced engagement in regular toothbrushing (OR/95% CI 0.475/0.384–0.587 for low health literacy; OR/95% CI 0.666/0.550–0.808 for medium health literacy).

**Conclusion:**

This study highlights the significant impact of gender, family affluence and health literacy on toothbrushing frequency amongst adolescents in Poland and Slovakia. The findings underscore the need for targeted oral health promotion strategies that consider gender differences, socioeconomic inequalities and the importance of health literacy in improving oral hygiene practises amongst adolescents.

## Introduction

Adolescent oral health has been recognised as a crucial factor in achieving overall well-being, impacting both physical, social, and psychological aspects of life (1, 2). The Global Oral Health Action Plan 2023–2030 seeks to address this issue by promoting early prevention, disseminating crucial information on combating health disparities, and developing innovative strategies to enhance oral health outcomes (3).

According to data from 2022 published by the World Health Organisation (WHO), untreated dental decay in children’s deciduous teeth affects nearly half of the population in Slovakia and Poland (4, 5). Untreated caries not only cause pain and infection but also strain families’ finances and healthcare systems worldwide. Furthermore, the aesthetic value of teeth can significantly impact individuals’ confidence and overall quality of life (6, 7).

Whilst individual behaviour, such as toothbrushing, was a primary focus of discussions encircling policy-making and prevention, a broader perspective now acknowledges the influence of social determinants. These, along with Common Risk Factor Approach (unhealthy diet, tobacco and alcohol consumption, physical inactivity and environmental pollution), significantly contribute to the development of non-communicable chronic diseases (NCDs) (8).

Self-care, the autonomous practise of maintaining health, is essential for well-being and oral disease prevention. Since most oral diseases are preventable, sustained self-care and informed choices by individuals and communities are key to improving oral health across the life course (9). Zaborskis et al. utilising data from 20 countries participating in the Health Behaviour in School-aged Children (HBSC) study between 1994 and 2018 examined cross-national trends in toothbrushing behaviour over time and its association with sociodemographic factors. The findings confirmed that gender and family affluence are the primary determinants influencing toothbrushing practises (10).

Health literacy (HL) significantly influences oral health by shaping individuals’ understanding of preventive care practises, improving their ability to make informed health decisions and encouraging behaviours that reduce the risk of dental issues (11, 12). Research indicates that the role of HL in achieving overall oral health is paramount, particularly given the limited oral health literacy amongst adolescent caregivers, which significantly impacts children’s oral health outcomes (13–16). In Central Europe disparities in oral health are often linked to socioeconomic factors, health literacy and access to preventive services. The number of dentist per capita differed significantly at 4.9 per 10,000 inhabitants in Slovakia and 3.5 per 10,000 inhabitants in Poland (4, 5).

Recent surge in focus on research of HL in Central Europe might be linked to Health Literacy Survey conducted in 2015 which shown poor level of health literacy in this region (17, 18). Although attempts have been made, the term of health literacy appears to not be the unified umbrella term the WHO presented in 1998, which states: “The cognitive and social skills which determine the motivation and ability of individuals to gain access to understand and use information in ways which promote and maintain good health” (13, 18, 19).

Therefore, the aim of the present study was first to assess the association between oral health and health literacy, gender, age, family affluence and country of origin amongst adolescents from two countries from the World Health Organization European region. In addition, this study sought to compare the associations between oral health and health literacy, gender, age and family affluence amongst adolescents from Slovakia and Poland separately. This analysis will provide insights into the effectiveness of current oral health strategies in both countries and potentially guide future public health initiatives. Our research study has been followed in accordance with the STROBE checklist and recommended guidelines for cross-sectional studies.

## Methods

### Sample and procedure

We used data from a Health Behaviour in School-aged Children (HBSC) study conducted in Slovakia and Poland in 2022.

In Slovakia, a two-step sampling was performed to obtain a representative sample. First, 195 larger and smaller elementary schools from rural and urban areas were randomly selected from a list of all eligible schools in Slovakia provided by the Slovak Institute of Information and Prognosis for Education and invited to participate. Ultimately, 94 schools agreed to participate (response rate: 48%). Second, data were collected from 9,697 adolescents in grades 5 to 9 of elementary schools in Slovakia using the HBSC survey (mean age 13.4 ± 1.3; 50.9% boys) from April to June 2022. Some of studied questionnaire data were administrated for adolescents older than 13 years, therefore the final sample consisted of 3,661 13- and 15-year-old Slovakian adolescents (mean age = 14.45; SD = 1.00; 52.6% boys). This study was approved by the Ethics Committee of the Medical Faculty of Pavol Jozef Šafárik University, Košice (13/N2021). Parents were informed of the study via the school administration and could opt out if they disagreed with their child’s participation. Children were informed about the study in advance by their teachers and again during data collection by the HBSC administrator, who explained the option to refuse participation. Participation was entirely voluntary and anonymous, with no explicit incentives offered. Data were collected through self-administered online questionnaires completed by pupils at schools’ computer classrooms during the classes, in the presence of researchers or research assistants.

In Poland, the HBSC study took place from the end of March to June 2022. A two-step sampling procedure was used to obtain a representative sample. Initially, 252 primary and secondary schools located in rural and urban areas from all 16 regions (voivodeships) of Poland were asked to participate. These were randomly selected from a list of all eligible schools in Poland obtained from the lists of schools and educational establishments of the Register of Schools and Educational Establishments (a module of the Educational Information System), which contains information on all currently operating or abolished schools and educational establishments in Poland. The school response rate (RR) was 64.7% (n = 163 schools). In the second step, data from 5,392 adolescents from the fifth and seventh grades of primary schools and the first grade of secondary school in Poland were obtained (mean age 13.87; 48.1% boys). The final sample consisted of 2,628 13- and 15-year-old Polish adolescents (mean age = 14.53; SD = 1.03; 47.6% boys). The online HBSC questionnaires were administered in the schools’ computer classrooms by trained teachers. The scope of the questionnaire and the procedure for organising the studies and obtaining consent from parents and students over the age of 13 to participate in the survey were reviewed and approved by the Bioethics Committee of the Institute of Mother and Child in Warsaw (opinion no. 51/2021 of 24 June 2021). Participation in the study was fully voluntary and anonymous, with no explicit incentives provided for participation. Students were given the opportunity to withdraw from the survey at any time during the survey.

The final combined Polish and Slovak sample consisted of 6,289 13- and 15-year-old adolescents (mean age = 14.48; SD = 1.01; 50.5% boys).

### Measures

Demographic data (*age, gender*) were collected using single questions that are used and validated in the HBSC surveys (20).

### Family affluence scale

Socioeconomic status was estimated using the Family Affluence Scale III (FAS-III), which consists of six questions: “Does your family own a car, van or truck?” (No / Yes, one / Yes, two or more); “Do you have your own bedroom for yourself?” (Yes / No); “How many computers does your family own?” (None / One / Two / More than two); “How many bathrooms (room with a bath/shower or both) are in your home?” (None / One / Two / More than two); “Does your family have a dishwasher at home?” (Yes / No); “How many times did you and your family travel out of your country for a holiday / vacation last year?” (Not at all / Once / Twice / More than twice). The responses to the items were calculated as an aggregated FAS index ranging from 0 to 13. We computed the sum score, which we converted to a ridit score ranging from 0 to 1. We then created tertile categories of low (0 to 0.333), medium (0.334 to 0.666) and high (0.667 to 1) socioeconomic position (20, 21).

### Health literacy amongst school aged children (HLSAC)

The purpose of the HLSAC is to examine adolescents’ perceived knowledge and competencies in making sound health decisions, as well as their ability to address and modify the factors that influence their own health and the health of others (21). Specifically, the HLSAC measures subjective and general health literacy; it is not a domain-specific measure (22). The HLSAC was internationally validated for 13- and 15-year-olds (22) and was developed for broader international use. The validated Slovak version of the HLSAC was used in our study (23). The HLSAC covers five core components: theoretical (health) knowledge, practical (health) knowledge, critical thinking, self-awareness, and citizenship (24) and includes two items for each core component. All the items took the form “I am confident that,” and the response options were (1) not at all true (2), not quite true (3), somewhat true and (4) absolutely true. A sum-score was generated out of the responses to its 10 items, ranging from 10 to 40. From the sum score, three levels (low, moderate, high) can be formed: low (score 10–26), moderate (score 27–35) and high (score 36–40), and the HLSAC was used with these discrete cut-off points in this study.

### Oral health

Toothbrushing was measured with a single frequency item: “How often do you brush your teeth?” with the five response categories (1: “More than once a day”; 2: “Once a day”; 3: “At least once a week but not daily,” 4: “Less than once a week” and 5: “Never”) (20). In the analyses for the present paper the outcome was defined as “toothbrushing more than once a day,” collapsing the four other categories to 0.

### Statistical analyses

Initially, we computed descriptive frequencies for gender, family affluence, country, age, health literacy and toothbrushing usage across the entire dataset, as well as specifically for the Polish and Slovakian subsets. Binary logistic regression was used to assess the associations of dependent variable - toothbrushing with the selected predictors. All independent variables were entered simultaneously into the models (enter method), based on theoretical considerations and prior research. Statistical significance was defined as *p* < 0.05. Results are presented as odds ratios (OR) with 95% confidence intervals (95% CI). The statistical analysis was conducted using IBM SPSS Statistics 21.0.

## Results

The frequencies and missing data of the whole sample are presented in Table 1.

**Table 1:** Descriptive characteristics for the whole sample.

Variable	Category	*N* (Valid %)
Gender	Boys	3,177 (50.5)
	Girls	3,112 (49.5)
Family affluence	Low	1,091 (19.5)
	Medium	3,317 (59.4)
	High	1,177 (21.1)
Country	Slovakia	3,661 (58.2)
	Poland	2,629 (41.8)
Age	13	3,731 (59.3)
	15	2,559 (40.7)
Health literacy category	Low	1,271 (26.7)
	Medium	2,797 (58.8)
	High	692 (14.5)
Toothbrushing	once a day and less	2,378 (38.8)
	more than once a day	3,744 (61.2)

Number of missing cases per variable: Gender—1; family affluence—705; country—0; age—0; health literacy—1,530; toothbrushing—168.

The frequencies and missing data of the Polish and Slovak data are presented in Table 2.

**Table 2:** Descriptive characteristics for the Polish and Slovak data sample.

Variable	Category	*N* (Valid %) Poland	*N* (Valid %) Slovakia
Gender	Boys	1,252 (47.6)	1925 (52.6)
	Girls	1,376 (52.4)	1736 (47.4)
Family affluence	Low	519 (20.3)	572 (18.9)
	Medium	1,437 (56.2)	1880 (62.1)
	High	602 (23.5)	575 (19.0)
Age	13	1,586 (60.3)	2,145 (58.6)
	15	1,043 (39.7)	1,516 (41.4)
Health literacy category	Low	708 (30.3)	563 (23.2)
	Medium	1,362 (58.3)	1,435 (59.2)
	High	265 (11.3)	427 (17.6)
Toothbrushing	Once a day and less	991 (38.3)	1,387 (39.2)
	More than once a day	1,596 (61.7)	2,148 (60.8)

Number of missing cases per variable Poland/Slovakia: Gender—1/0; family affluence—71/634; country—0/0; age—0/0; health literacy—294/1236; toothbrushing—42/126.

Table 3 presents the results of the binary logistic regression for regular toothbrushing (brushing the teeth more than once a day) in the combined sample of Slovak and Polish adolescents, adjusted for age, gender, family affluence, health literacy, and country of origin. Overall, significant associations were observed for gender, family affluence, and health literacy. Boys reported substantially lower odds of brushing their teeth more than once a day compared with girls. Similarly, adolescents from families with lower or medium affluence, as well as those with lower or medium health literacy, also showed significantly lower odds of regular toothbrushing compared with their peers from high-affluence families or with high health literacy.

**Table 3:** Associations of toothbrushing with age, gender, family affluence scale (FAS), health literacy and country: Odds ratios (OR) and 95% confidence intervals (95% CI) from binary logistic regression.

Variable	Category	Toothbrushing
		OR (95% CI)
Age	13	1.053 (0.929–1.194) ^ns^
	15	1 (ref.)
Gender	Girls	1 (ref.) ***
	Boys	0.431 (0.381–0.489) ***
FAS	Low	0.486 (0.399–0.592) ***
	Medium	0.761 (0.647–0.895) **
	High	1 (ref.) ***
Health literacy category	Low	0.475 (0.384–0.587) ***
	Medium	0.666 (0.550–0.808) ***
	High	1 (ref.) ***
Country	Slovakia	1 (ref.)
	Poland	1.024 (0.903–1.161) ^ns^
Negelkerke R square		0.086

***p < 0.001; **p < 0.01; *p < 0.05; ns, not significant; ref., reference group; FAS, family affluence scale.

Table 4 presents the binary logistic regression results comparing Polish and Slovak adolescents with regard to toothbrush usage (brushing the teeth more than once a day), adjusted for age, gender, family affluence, and health literacy. In both countries, boys reported significantly lower odds of brushing their teeth more than once a day compared with girls. Lower and medium family affluence were also consistently associated with less frequent toothbrushing in both samples. Similarly, adolescents with lower or medium health literacy had significantly lower odds of brushing their teeth regularly compared with those with high health literacy. Overall, the direction and strength of associations were comparable between the Polish and Slovak samples.

**Table 4:** Associations of the toothbrushing and age, gender, the family affluence scale (FAS) and health literacy for the Polish and Slovak samples: Odds ratios (OR) and 95% confidence intervals (95% CI) from binary logistic regression.

Variable	Category	Toothbrushing Poland	Toothbrushing Slovakia
		OR (95% CI)	OR (95% CI)
Age	13	1.146 (0.958–1.372) ^ns^	0.973 (0.815–1.161) ^ns^
	15	1 (ref.)	1 (ref.)
Gender	Girls	1 (ref.) ***	1 (ref.) ***
	Boys	0.451 (0.378–0.538) ***	0.412 (0.344–0.492) ***
FAS	Low	0.506 (0.388–0.661) ***	0.462 (0.343–0.622) ***
	Medium	0.775 (0.621–0.968) *	0.744 (0.585–0.946) *
	High	1 (ref.) ***	1 (ref.) ***
Health literacy category	Low	0.447 (0.324–0.616) ***	0.491 (0.369–0.654) ***
	Medium	0.625 (0.461–0.847) **	0.701 (0.546–0.901) **
	High	1 (ref.) ***	1 (ref.) ***
Negelkerke R square		0.085	0.092

***p < 0.001; **p < 0.01; *p < 0.05; ns, not significant; ref., reference group; FAS, family affluence scale.

The area under the ROC curve (AUC) indicated modest discriminatory performance of the models, with values of 0.648 for the total sample, 0.649 for the Polish subsample, and 0.652 for the Slovak subsample.

## Discussion

The aim of this study was to examine how gender, age, family affluence, and health literacy are associated with toothbrushing frequency amongst adolescents in Poland and Slovakia. Using data from the 2022 Health Behaviour in School-aged Children (HBSC) survey, collected via anonymous self-administered questionnaires in school settings, we explored the complex interplay of sociodemographic and cognitive factors in two Central European countries. Although differing in population size and certain structural aspects, Poland and Slovakia share a comparable post-socialist historical background, which provides a meaningful context for cross-national comparison. In addition to individual-level determinants, the findings invite further reflection on how national health governance structures and policy approaches may contribute to variations in oral health behaviours amongst adolescents.

The results indicate that boys are substantially less likely to engage in regular toothbrushing compared to girls, highlighting a persistent gender disparity in oral hygiene behaviours. Additionally, lower socioeconomic status, as measured by family affluence, is associated with a decreased likelihood of frequent toothbrushing, suggesting that economic factors play a crucial role in oral health practises. Similarly, health literacy emerges as a key determinant, with adolescents exhibiting lower health literacy levels demonstrating significantly reduced engagement in regular toothbrushing.

In recent years, Slovakia has proposed several initiatives, such as introducing a tax on sugar-containing beverages and mapping vulnerable social groups (25). Additionally, efforts are being made to include health workers in school workflows to educate children on oral hygiene and the importance of a balanced diet (26, 27). Furthermore, *the Slovak Dental Association (Slovenská komora zubných lekárov, SKZL)* has prioritised the promotion of oral health-related information specifically tailored for practitioners and parents (28). Additionally, insurance companies have introduced subsidised care packages covering dental treatment as well as preventive hygiene procedures (25).

Poland and Slovakia have adopted significantly different approaches to addressing adolescent oral health from both legislative and practical perspectives. In Poland, several local and governmental initiatives have been implemented, including the deployment of mobile dental clinics in underserved areas and the promotion of oral health prevention amongst vulnerable groups such as pregnant women and school-aged children (29–32). Although data on their impact is limited, the available information primarily focuses on general recommendations rather than targeted interventions tailored to community needs (33). In contrast, whilst Slovakia has outlined key goals for improving oral health, most initiatives are driven by private companies promoting health-related projects or by student-led efforts (34, 35). Furthermore, essential tools for assessing inequalities in access to dental care remain insufficient. Although basic oral healthcare for children is fully subsidised in both countries (36, 37), the number of accessible procedures appears to be disproportionately low compared to the actual needs of young patients (33, 38). Both countries have issued recommendations grounded in epidemiological data and supported by available preventive measures; however, to the best of the author’s knowledge, no clear implementation strategy has been established, reflecting a gap between expert guidance and political action (29, 35). Notable setbacks have also been observed in both countries’ oral care frameworks, such as the discontinuation of school-based dental care programmes in Poland and the cessation of supervised tooth-brushing in Slovak kindergartens—changes that are likely to have a significant negative impact on oral health outcomes (27, 39).

Identifying gaps in healthcare programmes in both countries is insufficient unless accompanied by a systematic approach to assessing and addressing the population’s specific needs. A health-literate system extends beyond the mere availability of services; it ensures that healthcare initiatives are accessible, comprehensible and effectively utilised by the target population. To achieve meaningful improvements in public health outcomes, the integration of structured needs assessments, policy-level commitments and continuous evaluation mechanisms that ensure that interventions are evidence-based, responsive and sustainable must be ensured (40).

Health literacy is crucial for individuals to navigate specific needs and follow proposed preventative measures. Future policy should prioritise education and mapping inequalities to meet personal healthcare needs. Simple preventive measures, like reading informative brochures or meeting specialists, are ineffective without proper education (41). In addition to personal health literacy, measures like the family affluence scale can indicate further disparities that need addressing (42). Although access to educational and self-directed resources is becoming increasingly widespread, this accessibility does not necessarily enhance the educational value for individuals seeking to understand these materials. In fact, it has been shown to be inefficient for patients with poorer educational outcomes (43). It is crucial to note that whilst many countries may prioritise the selection of social media or mobile applications in promoting health-oriented behaviours, it is essential to recognise that this approach may not necessarily align with the needs of the addressed population (44). This highlights the need for individualised care and tailored interventions to address the specific health needs of different populations.

Collaborative efforts amongst countries with similar socioeconomic contexts, such as the initiative amongst four Balkan countries (Albania, Bosnia and Herzegovina, Croatia and Serbia), are vital for addressing adolescent oral health disparities. However, the limited information on the implementation and outcomes of these strategies calls for further research into their effectiveness (45). A dialogue amongst oral health professionals could provide valuable insights into effective prevention strategies, drawing on experiences from countries such as Denmark, which has successfully reduced caries rates, and on practical resources like the preventive toolkits developed in the United Kingdom. Comparative research across countries provides valuable insights into weaknesses within health-care systems and highlights opportunities for strengthening NCDs prevention (46). Such exchanges could also inform future collaborative programmes in Poland and Slovakia, supporting the development of more effective, evidence-based approaches to improving oral and general health.

### Strengths and limitations

The main strengths of this study are its large sample size and the representative national datasets of two European countries. However, some limitations must be discussed as well. A first limitation is the cross-sectional design of our study, which hinders conclusive inferences about causality. An important limitation of our study is the relatively high proportion of missing data for some variables, especially family affluence (24.3%) and health literacy (11.2%). Because we relied on listwise deletion, the exclusion of these cases may have reduced statistical power and introduced bias if the missingness was related to participant characteristics. Subjective self-reports were used to measure the level of toothbrushing, which might be considered as a limitation. However, anonymity, confidentiality and privacy were all provided by the self-administration of questionnaires in the absence of teachers; this decreased the probability of the over- or under-reporting of health-related behaviour amongst adolescents, although some degree of social desirability or recall bias cannot be excluded. As this study relied on self-administered questionnaires, no clinical data such as dental history or other relevant risk factors (e.g., access to dental care, smoking, or alcohol use) were collected. Including such information would have provided a more comprehensive understanding of the interplay between health literacy and oral health outcomes. Future research should therefore integrate these variables within the common risk factor approach to better inform preventive strategies and policy development.

### Implications

The findings of this study highlight critical gaps in oral health behaviours amongst adolescents in Poland and Slovakia, underscoring the need for targeted interventions addressing gender disparities, socioeconomic inequalities and health literacy. These results have important implications for both research and practise. Examples may include school-based oral health education tailored to adolescents, gender-sensitive approaches to promote preventive practises, and subsidised preventive care for socioeconomically disadvantaged groups.

From a research perspective, future studies should focus on evaluating the effectiveness of existing oral health initiatives in both countries, particularly in relation to their impact on vulnerable populations. Given the differences in legislative and practical approaches to adolescent oral health, comparative studies assessing policy effectiveness, implementation strategies and long-term outcomes are necessary. Additionally, further investigation is needed to explore the underlying behavioural and structural barriers that contribute to lower toothbrushing frequency amongst boys, adolescents from lower socioeconomic backgrounds and those with limited health literacy (47, 48). Research should also incorporate qualitative methodologies to gain deeper insights into adolescents’ perceptions of oral health and access to care.

From a practical perspective, the study underscores the need for evidence-based, structured and collaborative approaches to oral health promotion. Whilst both Poland and Slovakia provide fully subsidised basic oral healthcare for children, the availability of dental procedures does not appear to meet actual demand. Policymakers should prioritise the development of comprehensive national strategies that integrate oral health education into broader public health initiatives. This includes enhancing school-based oral health programmes, fostering intersectoral collaboration between governmental and non-governmental organisations and ensuring that interventions are culturally and contextually appropriate.

## Conclusion

The findings of this study demonstrate that toothbrushing frequency amongst Polish and Slovak adolescents is strongly associated with gender, family affluence, and health literacy. By identifying these key sociodemographic and cognitive determinants, the findings provide evidence for public health practitioners and policymakers to better understand oral hygiene behaviours in adolescents and to develop population-level interventions that target the groups at greatest risk of inadequate oral care.

## Data Availability

The raw data supporting the conclusions of this article will be made available by the authors, without undue reservation.
